# Differential Spleen Remodeling Associated with Different Levels of Parasite Virulence Controls Disease Outcome in Malaria Parasite Infections

**DOI:** 10.1128/mSphere.00018-15

**Published:** 2015-12-09

**Authors:** Ximei Huang, Sha Huang, Lai Chun Ong, Jason Chu-Shern Lim, Rebecca Joan Mary Hurst, Annals Tatenda Mushunje, Paul Thomas Matsudaira, Jongyoon Han, Peter Rainer Preiser

**Affiliations:** a School of Biological Science, Nanyang Technological University, Singapore; b Department of Electrical Engineering and Computer Science, Massachusetts Institute of Technology, Cambridge, Massachusetts, USA; c Department of Biological Engineering, Massachusetts Institute of Technology, Cambridge, Massachusetts, USA; d Biosystems and Micromechanics IRG, Singapore-MIT Alliance for Research and Technology, Singapore; e Center for BioImaging Sciences, National University of Singapore, Singapore; f Department of Biological Sciences, National University of Singapore, Singapore; g MechanoBiology Institute-Singapore, National University of Singapore, Singapore; h Clinical Imaging Research Center, National University of Singapore, Singapore; University at Buffalo

**Keywords:** malaria, innate immunity, red blood cell rigidity, virulence, marker

## Abstract

Infections by malaria parasites can lead to very different clinical outcomes, ranging from mild symptoms to death. Differences in the ability of the spleen to deal with the infected red blood cells (iRBCs) are linked to differences in virulence. Using virulent and avirulent strains of the rodent malaria parasite *Plasmodium yoelii*, we investigated how parasite virulence modulates overall spleen function. Following parasite invasion, a difference in parasite virulence was observed in association with different levels of spleen morphology and iRBC rigidity, both of which contributed to enhanced parasite clearance. Moreover, iRBC rigidity as modulated by the spleen was demonstrated to correlate with disease outcome and thus can be used as a robust indicator of virulence. The data indicate that alterations in the biomechanical properties of iRBCs are the result of the complex interaction between host and parasite. Furthermore, we confirmed that early spleen responses are a key factor in directing the clinical outcome of an infection.

**IMPORTANCE** The spleen and its response to parasite infection are important in eliminating parasites in malaria. By comparing *P. yoelii* parasite lines with different disease outcomes in mice that had either intact spleens or had had their spleens removed, we showed that upon parasite infection, the spleen exhibits dramatic changes that can affect parasite clearance. The spleen itself directly impacts RBC deformability independently of parasite genetics. The data indicated that the changes in the biomechanical properties of malaria parasite-infected RBCs are the result of the complex interaction between host and parasite, and RBC deformability itself can serve as a novel predictor of clinical outcome. The results also suggest that early responses in the spleen are a key factor directing the clinical outcome of an infection.

## Abstract (precis)

The spleen and its response to parasite infection are important in eliminating parasites in malaria. By comparing *P. yoelii* parasite lines with different disease outcomes in mice that had either intact spleens or had had their spleens removed, we showed that upon parasite infection, the spleen exhibits dramatic changes that can affect parasite clearance. The spleen itself directly impacts RBC deformability independently of parasite genetics. The data indicated that the changes in the biomechanical properties of malaria parasite-infected RBCs are the result of the complex interaction between host and parasite, and RBC deformability itself can serve as a novel predictor of clinical outcome. The results also suggest that early responses in the spleen are a key factor directing the clinical outcome of an infection.

## INTRODUCTION

Malaria has been affecting humans for over 50,000 years (1). In spite of numerous measures that have been introduced to fight malaria, the disease persists as a major threat to global health. Over the past decades, increasing resistance to antimalaria drugs has been reported, and the prospect of having no effective cure to malaria is looming. It is therefore important to confront the old problem of malaria from a different perspective and explore new strategies to tackle this disease before we run out of options.

Malaria is broadly categorized as either uncomplicated or severe (i.e., complicated) by the WHO. Malaria severity is often linked with the parasite’s intrinsic virulence (2). *Plasmodium* spp. parasites display significant variations in their virulence, with some parasite strains and species being avirulent and easily cleared, whereas others trigger severe disease conditions that can kill the host. For example, in human malaria, *Plasmodium falciparum* and *P. knowlesi* can cause rapidly progressive severe illness or death, while other parasite species, like *P. vivax*, *P. ovale*, or *P. malariae*, are less likely to cause severe manifestations (3–5). Studies also indicate that red blood cell (RBC) retention in the spleen can modulate parasite virulence and control disease outcome (6).

Variations in drug resistance patterns of different parasite strains across different geographic regions (7, 8), along with the fact that malaria pathology can vary not only across species but also between different strains, create particular challenges in disease management. While patients with uncomplicated diseases can be effectively treated by oral antimalarials, patients with complicated conditions should be hospitalized and treated with parenteral antimalaria therapy. Diagnostic tools that can predict the risk of developing severe disease could significantly reduce the cost of disease management and allow the application of the best treatment options.

The spleen serves as a blood filter, as it monitors circulating pathogens, and it plays a central role in malaria parasite clearance (9). The importance of the spleen in malaria pathogenesis has been confirmed in a number of rodent and human studies (10–14). During replication of the asexual blood-stage parasite in the host, the spleen is the main organ that takes part in establishing the immune response and eliminating the parasites (15). In response to parasite infection, the spleen undergoes a series of morphological changes, the most apparent being the enlargement of the spleen, commonly referred to as splenomegaly. In fact, splenomegaly has been used as a clinical marker to estimate malaria transmission (16). Besides volume enlargement, the spleen also exhibits structural disorganization and remodeling, including the expansion of the red pulp, transient loss of the marginal zone, increased vasculature, and activation of barrier cells that could potentially lead to formation of the blood-spleen barrier and changes in the splenic blood circulation (17–20). In addition to the conventional views of the spleen as an important immune effector, the splenic clearance of the parasite is also seen as a highly “mechanical” process. In fact, the spleen is a very complex organ with a complicated vascular arrangement and thus a complicated microcirculatory network. Blood enters the spleen through the splenic arteries, which branch to trabecular arteries and further branch to central arterioles. In humans, in which the majority of the splenic blood traverses directly into the venous network, bypassing the red pulp, the remaining blood empties into the red pulp filtration bed before being drained out by the venous system in a slow open circulation (21). The reticular meshwork in the red pulp mechanically challenges the blood cells such that old or abnormal erythrocytes, which are less deformable, are likely to be retained and eventually destroyed (22). Indeed, RBC mechanical retention in the spleen is believed to be one key mechanism facilitating the removal of infected erythrocytes during malaria parasite infection. Parasitized erythrocytes with compromised deformability are more likely to be retained in the red pulp before destruction. Conversely, transformations in the red pulp and splenic vasculature may modulate the mechanical retention threshold and regulate the microcirculatory trapping of blood cells in the spleen. Increased splenic retention of infected erythrocytes assists the clearance of parasites, lowering the risk of severe malaria. However, excessive blood retention could lead to malaria anemia and is therefore undesirable (21).

RBC deformability in relation to malaria pathogenesis has been discussed extensively (23–26). In general, infection with the parasites results in decreased RBC deformability, which has been demonstrated through different methods, including use of ektacytometry (27), micropipette aspiration (28), laminar flow systems (29), optical tweezers (30), and microfluidic devices (31). Though the mechanisms underlying the changes in cell deformability during *Plasmodium* infection are poorly understood, reduced RBC deformability may contribute to impaired blood microcirculation and organ dysfunction (32). Much evidence has also indicated that, besides ligand-receptor interactions, reduced RBC deformability is greatly associated with splenic clearance (21, 29, 33). Indeed, splenic retention of parasitized RBCs starts very early during the infection and may help to activate the antigen-specific responses (26). In addition, data from *in vitro* studies and field reports have suggested an association between RBC deformability and disease severity (27, 29, 34, 35). However, there is, so far, no direct evidence to show the correlation among cell deformability, spleen remodeling, and infection severity.

Here, we attempt to gain a better understanding of malaria pathogenesis through increased comprehension of how differences in parasite virulence and differential spleen responses lead to diverse disease outcomes. Since there are ethical and technical constraints on the study of the human spleen in malaria, most of the known information has been obtained indirectly from *ex vivo* models. However, spleen remodeling is a dynamic and complicated response, and *ex vivo* studies alone do not provide sufficient information to understand disease development and predict disease outcome. On the other hand, though there are anatomical differences between human and mouse spleens, many morphological features of the spleen are conserved (36). In addition, the rodent malaria model organism *P. yoelii* has long been employed in malaria research to complement the research on human malaria. The existence of parasite clones of *P. yoelii* with different RBC preferences, growth behaviors, and virulence, that together result in very different clinical outcomes in BALB/c mice, greatly facilitates research on different aspects of human malaria diseases. Strains like YM and 17XL invade RBCs of all ages and cause a lethal infection, while strains like YA and 17X1.1 preferentially invade reticulocytes and result in a self-limiting infection that is eventually cleared by the host (37, 38). It has been shown that different parasite strains stimulate the remodeling of the spleen differently (20, 39). In this study, we investigated in detail the remodeling of the spleen and its relationship with RBC deformability, parasite virulence, and clinical outcome. Our data show that *Plasmodium* infections stimulate dramatic and differential splenic responses in infections with different disease outcomes. Moreover, we show that changes in infected RBC (iRBC) deformability correlate strongly with clinical outcome and that, crucially, the spleen itself directly impacts iRBC deformability independently of the parasite strain. Finally, we provide data that show that RBC deformability can be directly utilized as a predictor of clinical outcome and therefore may serve as a diagnostic marker for malaria prognosis. In summary, our findings not only introduce a new insight into the understanding of the pathogenesis of malaria but also provide a simple and fast alternative method for accurately predicting malaria disease outcome to aid the early decisions undertaken by medical staff for effective treatment of malaria.

## RESULTS

### Presence of the spleen protects the host in a mixed-strain infection model.

**FIG 1:**
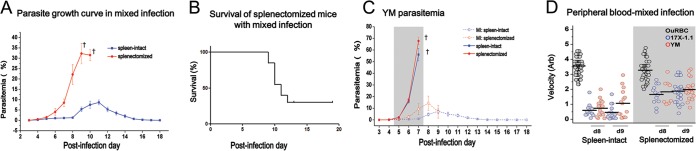
Results with the mixed-infection model with *Plasmodium yoelii*. (A) Parasite growth curve for total parasites after mixed infection of mice with intact spleens and splenectomized mice. Data are mean (± standard error of the mean [SEM]) results from four individual experiments, each with 3 to 7 mice per group. The † symbols denote animal deaths. (B) Survival curve for splenectomized mice in the mixed-infection group. Data were pooled from 20 mice in four individual experiments. (C) Parasite growth curve for YM in mixed infections (MI) as well as single YM infection of mice with spleens intact or splenectomized mice. Data are mean (± SEM) results from four individual experiments each with 3 to 7 mice per group. The † symbols denote animal deaths. (D) Deformability/velocity of RBCs in the mixed-infection model on different days postinfection. The lines indicate the mean values of each population of RBCs. Data were pooled from two individual experiments each with three mice per group.

Previous studies have demonstrated that infection with the *P. yoelii* YM line is lethal while the *P. yoelii* 17X line is nonlethal (38), and avirulent parasite strain infections can protect the host from challenge with virulent parasite infection (40). Here we used parasite lines expressing the markers green fluorescent protein (GFP) or mCherry. These parasites have distinct growth profiles and disease outcomes in single infections (see Fig. S1 in the supplemental material) that are very similar to those of infections with wild-type parasite lines (41). However, in a mixed-infection model, where mice were preinfected with the avirulent 17X line and challenged 24 h later with the virulent YM line, the total parasitemia increased very slowly. Indeed, the peak parasitemia was only around 10% on day 11 postinfection (p.i.), and parasites were cleared by day 17 p.i. (Fig. 1A). This protective effect applies only for mice with an intact spleen; in splenectomized mice, the majority developed high parasitemia close to 30% on day 8 p.i. (Fig. 1A) and started to die from day 9 p.i. onwards (Fig. 1B). However, compared to the single-parasite infection model, we noted that for both hosts with intact spleens and splenectomized hosts, the parasitemia of YM-infected cells in the mixed-infection model was significantly lower (Fig. 1C). For example, in the mixed-infection model, the peak YM parasitemia was suppressed to around 7% and 15% in mice with spleens and splenectomized mice, respectively.

10.1128/mSphere.00018-15.1 Figure S1  *Plasmodium yoelii* growth behavior in BALB/c mice. (A) Growth curve for the YM-GFP line in mice with intact spleens and splenectomized mice. Data are presented as means ± standard errors of the means (SEM) of results from three individual experiments, each with 5 mice per group. The † symbols denote deaths of mice. (B) Growth curve for the YM-mCherry line in mice with intact spleens and splenectomized mice. Data are means ± SEM results from three individual experiments, each with 5 mice per group. (C) Growth curve for the 17X1.1-GFP line in mice with intact spleens and splenectomized mice. Data are means ± SEM results from four individual experiments, each with 4 to 5 mice per group. (D) Survival curve for splenectomized mice infected with the 17X1.1-GFP line. Data were pooled from 19 mice in four individual experiments. Download Figure S1, TIF file, 0.8 MB. Copyright © 2015 Huang et al. 2015 Huang et al. This content is distributed under the terms of the Creative Commons Attribution 4.0 International license.

Changes in RBC deformability were monitored in parallel, as indicated by the transit velocity of individual cells when measured by a microfluidic device. A low RBC velocity corresponds to impaired cell deformability, and vice versa. Regardless of the presence of a spleen, the uninfected RBCs (uRBCs) were consistently more deformable than iRBCs. In particular, iRBCs from the mice with intact spleens were close to 2-fold less deformable than those from splenectomized hosts (Fig. 1D).

### Avirulent parasite infection corresponds to further impairment in iRBC deformability, leading to greater spleen retention.

**FIG 2:**
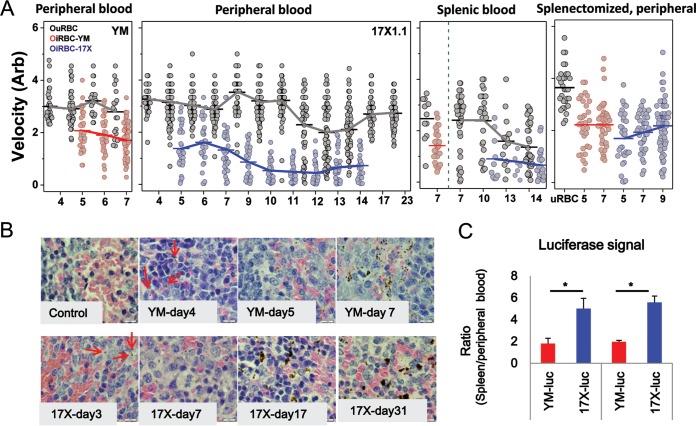
Changes in RBC deformability and spleen retention of iRBCs. (A) Deformability/velocity of RBCs in mice with parasite infection on different postinfection days. iRBC-YM, YM-GFP-infected RBCs; iRBC-17X, 17X1.1-GFP-infected RBCs. The lines connect the mean results of each RBC population. Data were pooled from at least three individual experiments, each with three mice per group. Statistical analysis using a one-way ANOVA with Tukey test was performed for the following comparisons: (i) iRBCs versus uRBCs; (ii) 17X- versus YM-infected peripheral blood samples and between day 5 versus day 7 p.i. results; (iii) peripheral blood, splenic blood, and splenectomized peripheral blood samples from YM-infected mice on day 7 p.i.; (iv) peripheral blood samples from mice with intact spleens versus splenectomized mice, both infected with 17X, at day 7 p.i. (B) H&E staining of spleen sections for different postinfection days under 100× magnification. Red arrows, parasite pigments. Representative images are presented (*n* = 5). (C) Comparison of parasite loads in peripheral blood and spleens for animals with different parasite infections. Data are means ± standard errors of the means of results from two individual experiments, each with three mice per group. *, statistically significant (*P* < 0.05).

To investigate whether the differences in iRBC deformability correlate well with different disease outcomes, we simultaneously monitored iRBC deformability, spleen remodeling, and parasitemia in single-infection models with different parasite virulence levels. Consistent with previous work, iRBCs were consistently stiffer than uRBCs in both the YM and 17X infection models (Fig. 2A). However, whereas the deformability of both iRBCs and uRBCs remained fairly stable across the postinfection days (days 4 to 7) for the YM-infected mice, RBC deformability varied significantly in 17X-infected mice. Specifically, 17X-infected hosts displayed significant reductions in iRBC and uRBC deformability, respectively, from day 7 and day 12 p.i. onwards (*P* < 0.05; one-way analysis of variance [ANOVA]). Furthermore, 17X-infected cells began to exhibit significantly lower deformability than did YM-infected cells during early infection days, when the parasitemia was under 1% (day 5 p.i.) (*P* < 0.05); the deformability of 17X-infected RBCs decreased further, by over 40% from day 9 p.i. onwards, until all detectable parasites were cleared (Fig. 2A).

The deformability of blood cells extracted from mouse spleens (i.e., splenic blood) was also assessed. In YM-infected mice, the mean velocities of both the infected and uninfected RBCs in the splenic blood were significantly lower than for RBCs in the peripheral blood (*P* < 0.05). Similarly, in 17X-infected mice, the splenic minced uninfected RBCs were less deformable than those in the peripheral blood (*P* < 0.05), but no significant change in velocity for the infected cells between the splenic blood and peripheral blood was observed. Furthermore, no further stiffening was observed when splenic versus peripheral infected cells were compared for day 10 to 14 p.i.

To further demonstrate the connection between RBC deformability and splenic cell retention, the deformability of peripheral blood RBCs from splenectomized mice was also measured. With spleen removal, the deformability of the infected cells between YM and 17X parasite lines was no longer significantly different except on day 5 p.i. (*P* = 0.036). The postinfection day-dependent decrease seen in 17X-infected RBC deformability was no longer observed either (Fig. 2A).

Our experiments showed clear differences in iRBC deformability between virulent and avirulent parasites lines, with the latter resulting in much lower iRBC deformability, corresponding to greater spleen retention. To confirm this, spleen sections were prepared and examined via bright-field microscopy coupled with hematoxylin and eosin (H&E) staining (Fig. 2B). Parasite pigments were observed 1 day earlier in mice infected with the avirulent 17X line (day 3) (Fig. 2B, red arrows) and present even after parasite clearance (noted on days 24 and 31 p.i.), indicating a preferential trapping of the 17X-infected RBCs compared to YM-infected RBCs. The parasite load reflected via a luciferase signal in peripheral blood as well as in the spleen was also assessed and compared using luciferase-conjugated parasite lines (Fig. 2C). Mice infected with the 17X line showed a significantly higher relative luciferase signal in the spleen than in the peripheral blood, compared to results in YM-infected mice on both days 4 and 5 p.i., confirming that 17X-infected RBCs are more preferentially trapped in the spleen. The lower deformability of the avirulent parasite is consistent with the earlier and higher parasite deposition observed in the spleen, indicating that mechanical retention of iRBCs in the spleen acts as a key contributor to parasite clearance.

### Varied splenic responses upon infections with different parasite.

**FIG 3:**
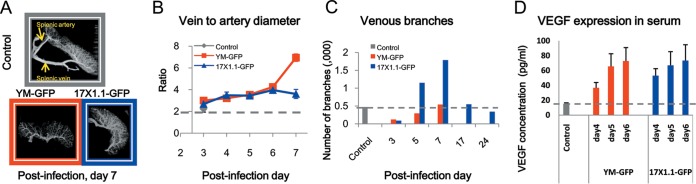
Changes in splenic architecture upon infection. (A) Reconstructed spleen cast images. Splenic vein and artery are shown by the yellow arrows (see also Fig. S2 in the supplemental material). (B) Ratios of vein to artery diameters. Data are means ± standard errors of the means (SEM) for results from the three main pairs of veins and arteries of one representative spleen cast of three samples per group. (C) Quantification of the venous branches in spleen casts. Data are from one representative spleen cast of three samples per group. (D) Expression of VEGF in mouse serum. Data are means ± SEM results from a total of five serum samples per group, collected in two replicate experiments.

Since changes in RBC deformability have been shown to be associated with spleen recognition and subsequent parasite clearance, the spleen responses upon parasite infection, in particular the changes in the splenic microcirculatory network, were also studied. Spleen plastination casts were prepared to examine changes in the circulation in the spleen upon infection. As shown in Fig. 3A and also Fig. S2 in the supplemental material, the splenic veins and arteries were identified and the internal vessels as well as the red pulp meshwork could be recognized. The complexity of splenic internal vessels, in terms of the number of venous branches and the relative sizes of veins and arteries, appeared to vary with parasite infection and parasitemia changes. Figure 3B is a plot of the splenic relative vein-to-artery diameter ratios during the course of disease development. The vein-to-artery diameter ratios increased gradually in both infection models, with a significant difference from the controls from day 4 p.i. onwards (*P* < 0.05). Moreover, the ratios in the YM infection model increased more significantly on day 7 p.i. than with 17X infection (*P* < 0.001). We noted that the diameters of splenic arteries remained relatively constant in both infection models throughout the postinfection days, and the significant increases in the ratio were largely due to dilation of the splenic veins upon infection (data not shown). The vein-to-artery ratio returned to normal after parasite clearance in mice infected with the avirulent 17X line (data not shown). In addition to the splenic vessel size, we also quantified the number of venous branches on different postinfection days. Figure 3C illustrates that more veins formed upon avirulent 17X infection than with virulent YM infection, and the number of venous branches returned to normal after parasite clearance. Systemic vascular endothelial growth factor (VEGF) levels in the serum were also measured (Fig. 3D), confirming that there were new vessels synthesized in response to parasite infection. We observed a significant increase in VEGF concentration upon parasite infection (*P* < 0.05), though there was no significant difference in the VEGF levels in the hosts infected with YM versus 17X. It is therefore not yet clear what molecular mechanism drives the observed differences in venous branch development between the virulent and avirulent parasite infections.

10.1128/mSphere.00018-15.2 Figure S2  Gallery of reconstructed images for spleen casts on different postinfection days. Download Figure S2, TIF file, 0.6 MB. Copyright © 2015 Huang et al. 2015 Huang et al. This content is distributed under the terms of the Creative Commons Attribution 4.0 International license.

### Deformability-based malaria prognosis.

**FIG 4:**
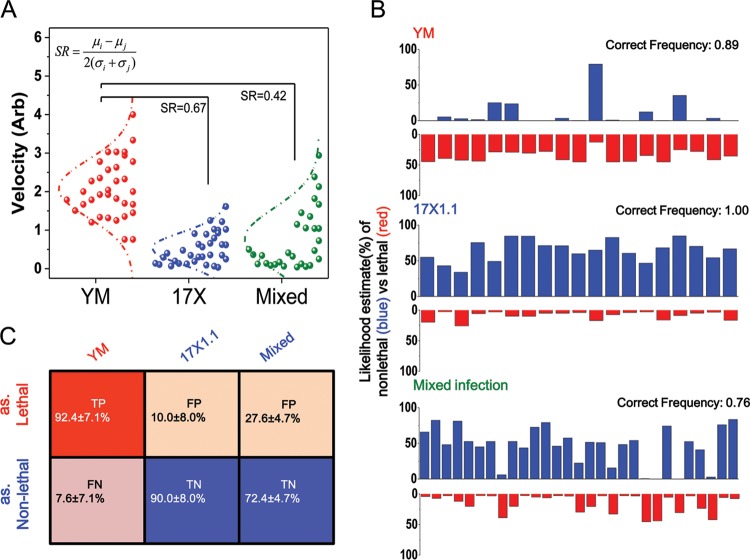
Prognosis estimation methods for malaria. (A) Fit of Gaussian curves for deformability profiles of respective infection models. (B) Illustration of MLE results after one subsampling. (C) Evaluation scores of the different infection models.

The substantial changes in cell deformability, along with the changes in the spleen microcirculatory network, allow more efficient clearance of the avirulent parasites, which probably accounts for the less severe disease outcome. The high correlation between RBC deformability and disease severity led us to explore the possibility of using deformability as a biomarker for a fast malaria prognosis. With a similar parasite load at around 5% parasitemia (i.e., YM at day 5 p.i., 17X at day 10 p.i., and mixed infection at day 9 p.i.), we first constructed Gaussian curves to fit the deformability profiles of the respective infection models (Fig. 4A). The mean (and standard deviation) of iRBC deformability from YM, 17X, and mixed-infection models were 2.08 ± 0.75, 0.5 ± 0.41, and 0.77 ± 0.79, respectively. Pairwise separation resolution (SR), which describes how far apart two given populations are from each other, was also calculated, such that the deformability SR values between YM- and 17X-infected RBCs and between YM- and mixed-model iRBCs were 0.67 and 0.42 (Fig. 4A). The formula we used to calculate separation resolution was as follows: SR_*i*,*j*_ = (μ_i_ − μ_j_)/2(σ_i_ + σ_j_), where μ_i_ and μ_j_ are the mean deformability values for RBCs infected with parasite infection models *i* and *j*, respectively, and σ_i_ and σ_j_ denote the standard deviations of deformability values in the respective infection models.

Since YM infection always induces a lethal disease outcome and 17X infection always leads to a mild clinical condition, we then estimated the severity of parasite infection by using the maximum likelihood estimation (MLE) based on the deformability of a given single iRBC alone. We randomly subsampled 15 out of 34 RBCs each from the YM and 17X infection groups and termed these samples the training set. The remaining 19 YM-infected, 19 17X-infected, and 29 mixed-infection model iRBCs (14 17X-infected RBCs and 15 YM-infected RBCs) were used as the testing set. Gaussian fittings, as described above, were then constructed using the training set, such that the deformability distributions of lethal and nonlethal infections were defined based on the 15 YM- and 17X-infected RBCs, respectively. We next calculated the probability densities of remaining RBCs in the test group against the training set and applied MLE to assign individual RBCs to the disease outcome in which they are more likely to fall into. Figure 4B illustrates the MLE result after one iteration of random sampling. Close to 90% of the YM-infected RBCs, 100% of the 17X-infected RBCs, and 76% of the mixed-model iRBCs, all from the testing data, were correctly classified into their corresponding clinical outcome groups. Note that in this evaluation, we considered the disease outcome of mixed infection as “nonlethal,” as almost all mice survived. After 20 similar iterations, our deformability data could identify a lethal infection at >90% true-positive (TP) and true-negative (TN) rates in single-infection models (Fig. 4C), achieving 91% predictive accuracy overall. Even in the mixed-infection model, where the deformability distribution was more complex, the MLE model could still produce a TN rate of 72%.

## DISCUSSION

The central function of the spleen is to selectively remove RBCs, microbes, and other particles from circulating blood, through mechanical trapping and/or the activation of the immune system. Malaria parasite infection stimulates dramatic spleen changes in both morphology and architecture. Although the virulent YM and the avirulent 17X lines have a similar genetic background, they have very different phenotypic characteristics, including parasite growth rate, red blood cell preference, surface protein expression, and stimulation of an immune response (37, 42, 43). The importance of splenic mechanical trapping for parasite clearance, as a contributing factor to such a phenotypic difference, is often overlooked. In this study, we took a new perspective and attempted to understand malaria pathology through spleen remodeling and retention. The essential role of the spleen in malaria pathogenesis was verified by examining the effect of splenectomy for hosts infected with either virulent or avirulent malaria strains. Removal of the spleen resulted in severe disease outcomes even for avirulent 17X parasite infection, in line with the spleen controlling parasite growth and determining disease outcome in nonlethal parasite infection. Distinct splenic responses during infection with different malaria parasite strains were also observed, such that 17X-infected mice displayed more prominent spleen remodeling, facilitating better parasite clearance. The earlier parasite deposition and greater preferential splenic iRBC trapping, as well as a greater impairment in iRBC deformability, were collectively observed in 17X-infected mice but not in YM-infection hosts. The strong correlation between iRBC deformability, spleen remodeling, and clinical outcome was further confirmed by our mixed-infection model, in which preinfection with the avirulent 17X line induced an “avirulent-like” spleen response and protected the mice from displaying severe clinical complications. Notably, in the mixed-infection model, the deformability of RBCs infected with the virulent parasite strain was decreased considerably, to a level similar to that in cells with avirulent parasite infections. Overall, our data support the novel prospect that the strain-specific spleen remodeling likely modulates pathological outcome withh malaria. Additionally, the deformability of iRBCs may be adopted as a potential disease marker, as differences in cell deformability were strongly associated with different disease outcomes.

While in both YM and 17X infections the spleen undergoes significant remodeling, it is clear from our data that in 17X infection there is a significant increase in the spleen size, venous pore size, and splenic vein-to-artery diameter ratio (Fig. 3; see also Fig. S3 in the supplemental material). This suggests that there is a significant increase in the overall blood volume that is filtered in the spleen and subsequently collected by the increased venous outflow in 17X infections. This finding, together with the decreased deformability of iRBCs in 17X infection, is consistent with increased retention of iRBCs as a key mechanism by which the spleen functions to control avirulent parasite infections.

10.1128/mSphere.00018-15.3 Figure S3  Changes in spleen size upon infection. (Upper panel) Selected images of spleens for different postinfection days. (Lower panel) Measurements of spleen length on different postinfection days. Red bar, YM-GFP-infected spleens; blue bar, 17X1.1-GFP-infected spleens. Data are means ± standard errors of the means (*n* = 5). *, statistically significant (*P* < 0.05). Download Figure S3, TIF file, 0.4 MB. Copyright © 2015 Huang et al. 2015 Huang et al. This content is distributed under the terms of the Creative Commons Attribution 4.0 International license.

Though there are some anatomical differences that have been identified between the human and mouse spleens, such as the presence of the perifollicular zone and the absence of the marginal sinus in the human spleen, they share many common properties of structure and function, including blood filtration (36). While the white pulp predominates in the mouse spleen but the red pulp is the largest compartment in the human spleen, and with the existence of the venous sinus, which provides an additional mechanical challenge to RBCs (36, 44), these structures potentially facilitate better blood filtration in the human spleen. However, these differences probably change the filtration degree but not the filtration function. Therefore, such differences between mice and humans would present a limited impact on interpretation of our data.

There are many factors that can contribute to altered cell deformability, including the size, shape, and viscoelasticity of the cell membrane, the state of the cytoplasm, and the fluidity of the hemoglobin (45). Upon infection, the presence of the intracellular *Plasmodium* parasite brings about cytoskeleton remodeling of the host cell, significantly impairing the deformability of RBCs (46). Our finding that the spleen and its response to infection regulate iRBC deformability was surprising. as it indicated that cellular external factors also impact deformability. One possible explanation is that specific factors, such as circulating antibodies that recognize the surface of iRBCs, could contribute to changes in deformability. However, with the early effects observed in our data, it is unlikely that specific antibodies are the main contributing factor, as antibodies are unlikely to play a role in an early stage of infection. On the other hand, since there is a large population of macrophages residing in the spleen, macrophage recognition may potentially influence the iRBC deformability. It has been shown that infection with the avirulent *P. yoelii* 17X line stimulates enhanced peritoneal macrophage activation compared to virulent strain infection (47). However, whether there is differential activation of the splenic macrophages between virulent and avirulent infections is not fully understood. In addition, whether the repeated passage through the microvasculature of the differently remodeled spleen or the host responses, such as early cytokine responses, drives the observed changes in deformability also needs to be established.

Interestingly, the presence of reticulocytes reduces the cell deformability, as they have significantly lower velocities than normocytes (see Fig. S4 in the supplemental material). However, parasite infection further increases reticulocyte rigidity by 2.4-fold (see Fig. S4). Therefore, the changes observed regarding cell deformability are unlikely due to the presence of reticulocytes alone.

10.1128/mSphere.00018-15.4 Figure S4  Deformability/velocity differences between uninfected reticulocytes and normocytes. (A) Deformability/velocity of reticulocytes and normocytes in uninfected mice. (B) Comparison of the deformability/velocity between uRBCs and iRBCs of 17X-infected RBCs in mice with more than 10% reticulocytes and parasitemia of around 1.5%. Download Figure S4, TIF file, 0.8 MB. Copyright © 2015 Huang et al. 2015 Huang et al. This content is distributed under the terms of the Creative Commons Attribution 4.0 International license.

It is often thought that the virulent YM parasites have a growth advantage compared to avirulent strains as a mechanism for strain-specific intrinsic virulence. However, this common presumption was clearly not supported in our mixed-infection model study, where the avirulent 17X parasite infection actually suppressed the growth of the virulent YM parasites. It is also counterintuitive to expect the combined peak parasitemia in the mixed-infection model to be lower than that in single-strain infections (Fig. 1; see also Fig. S1 in the supplemental material). The observed slow parasite growth and overall nonlethal disease outcome, however, correlated well with the low deformability values of RBCs infected with either the YM or 17X lines. In fact, the circulating RBCs in the mixed-infection model exhibited similar deformability as in the 17X infection model. This observation suggests the potential of RBC deformability as a novel, important marker to predict the disease severity of malaria (Fig. 4).

When a patient is infected with a malaria parasite, it is difficult to make an early-stage prognosis on whether the infection will be lethal or nonlethal. According to the WHO guidelines, a patient would first be given standard oral treatment, unless further complications develop. The prospect of performing early-stage lethal infection prediction and applying differential antimalarial treatment would be of great medical and economic value. Here, we have demonstrated that iRBC deformability can be used as a robust predictor of clinical outcome. Based on just single iRBCs, we could predict infection severity with an overall accuracy of over 90% for the single-infection model. In fact, we would argue that all iRBCs follow approximately an identical independent distribution (IID), and with our high-throughput microfluidic platform and its capability of measuring hundreds of RBCs within minutes, we may have a good chance of predicting infection lethality with further improved accuracy and making decisions through comparing more similar blood samples very early postinfection, when parasitemia is even lower.

In conclusion, we examined the differential splenic responses upon virulent and/or avirulent malaria parasite infection and identified a strong association across cell deformability, spleen remodeling, and disease severity. Infection induced dramatic changes in the spleen, including enlarged spleen sizes, dilated venous vessels, and increased venous branches; all of these changes support the notion that the spleen can function as an efficient “mechanical filter” with its adjustable meshwork. Evidence for splenic trapping of parasitized RBCs was further corroborated by the observation of early parasite deposition in the red pulp. We noted that avirulent parasite infection induced greater structural changes in the spleen, which contributed to better blood filtration. Furthermore, changes in RBC deformability upon parasite infection and during the course of disease development were also noted. Removal of the spleen resulted in increased deformability of RBCs in circulating blood upon infection with the avirulent 17X parasites, highlighting the role of the spleen in modulating cellular mechanical properties. Reduced RBC deformability correlates with more efficient parasite clearance. Parasitized RBC deformability is therefore, for the first time, proposed to be an important marker for early-stage prognosis of the virulence of parasite infection. As a simple demonstration, our MLE model achieved over 90% prediction accuracy when the host had a single-strain parasite infection. This provides a potential tool for fast and easy diagnosis and prognosis for malaria patients, helping to make a rapid response in choosing the correct treatment.

## MATERIALS AND METHODS

### Parasite preparation and infection.

Male BALB/c mice, 6 to 8 weeks old, were obtained from InVivos Singapore and subsequently bred under specific-pathogen-free (SPF) conditions at the Nanyang Technological University Animal Holding Unit. Mice were infected with cryopreserved stocks of different *P. yoelii* lines, and parasitemia was monitored daily from day 3 p.i. onwards by flow cytometry. Schizont-stage parasites were separated and cultured as previously described (48), and a standard inoculation of 1,000 mature infected cells was injected into new mice by intravenous (i.v.) injections. In the mixed-infection model, mice were infected with avirulent 17X1.1-GFP parasites and challenged with virulent YM-mCherry parasites 24 h later.

### Mouse splenectomy.

Mice of 4 to 5 weeks of age underwent splenectomy. Mice were anesthetized with a ketamine and xylene mixture by intraperitoneal (i.p.) injection and then given the painkiller meloxicam (0.2 mg/kg of body weight) i.p. The entire spleen was gently removed. One milliliter of phosphate-buffered saline (PBS) was injected i.p., and antibiotics (Septrin) were provided in drinking water continuously for 3 days after the surgery. Mice were kept in individual cages for another 10 to 15 days for recovery, and after that the mice were used for experimental infection.

### Spleen histology.

To assess spleen morphological changes in response to parasite infection, mouse spleen samples were prepared for histology. Mice were euthanized, and the spleen was carefully removed and immersed into 10% buffered formalin (Sigma). The samples were then processed for paraffin embedding, sectioning, and H&E staining. Images were captured using bright-field microscopy under 100× magnification.

### Parasite bioluminescent assay.

To quantify the parasite load in the spleen, genetically modified parasite lines expressing GFP-luciferase throughout the parasite life cycle were generated and used for the infection. Similarly, mice were infected with 1,000 mature infected cells and subjected to a bioluminescent assay on different postinfection days. Briefly, mice were injected with luciferin (Caliper) i.p., and the *in vivo* peripheral luciferase signal was detected by using an IVIS imaging system (PerkinElmer). Mice were then anesthetized for whole-body perfusion with 1× PBS. The spleens were removed, and the spleen luciferase signal was again detected with the IVIS imaging system. The ratios of the luciferase signal of the spleen to that in peripheral blood for each mouse were calculated and compared.

### Spleen corrosion cast preparation and analysis.

To study the spleen vascular changes upon parasite infection, spleen casts were prepared. Mice were euthanized with preinjection of heparin. A 22-gauge catheter (BD) was surgically inserted into the descending aorta. The spleen was first perfused with 1× PBS followed by complete Mercox II solution (Ladd Research Industries) injected through the catheter. Mice were left overnight for Mercox II polymerization and then were immersed into 10% NaOH for tissue digestion for a period of 10 to 14 days. Spleen samples were washed with distilled water, and spleen casts were air dried and collected for analysis.

The spleen casts were scanned using an Inveon CT scanner (Siemens). The scan was acquired with 2-by-2 binning at an exposure time of 7,800 ms per projection. A total of 1,200 projections for high-resolution and high-magnification image scans were performed. The acquired images were assembled using Inveon software and then processed, reconstructed, and visualized using MatLab. The vessel complexities were analyzed using FIJI software (49). Diameters of splenic veins and splenic arteries at the points where they connect with the spleen were measured, and the numbers of venous branches were quantified.

### Serum VEGF determinations.

To examine the vascular changes upon parasite infection, mouse serum was prepared and VEGF levels were measured and compared using a sandwich enzyme-linked immunosorbent assay (ELISA) according to the manufacturer’s protocol (eBioscience).

### Red blood cell deformability measurements using a microfluidic device.

To investigate the mechanical properties of the RBCs, a microfluidic deformability device was used as previous described (50). Prior to each experiment, the device was first mounted to a microscope, flushed with RPMI 1640 with 20% fetal bovine serum (complete medium), and incubated at room temperature for 20 min. Meanwhile, freshly collected mouse blood was diluted with complete medium to reach a final hematocrit between 0.1 and 1%. In detail, peripheral blood was collected from the mouse tail, while splenic blood was squeezed from the spleen after washing twice with 1× PBS. Blood samples (3 µl) were loaded into the device reservoir, and a low pressure was applied, as described previously. The deformation of RBCs in the microfluidic channel was captured using a charge-coupled-device camera, and all videos were analyzed using ImageJ. RBC velocity was defined as the horizontal displacement as RBCs traverse the device divided by the time in terms of the number of frames taken.

### Maximum likelihood estimation.

For the maximum likelihood estimation (MLE), we let Θ denote the two possible infection outcomes (i.e., lethal or nonlethal infection) and *x* denote the observed RBC deformability. The likelihood function *L* is then represented simply as the probability density of the observed deformability *x* at a specific infection state (i.e., disease outcome). Mathematically, the calculations were as follows: Θ = {lethal, nonlethal} and *L*(θ;*x*) = *f*(*x*|θ). Then, the MLE function of θ(^) (“theta hat”) is simply θ(^) = arg max_θ∈Θ_ *L*(θ;*x*).

### Statistics analysis.

Statistical significance was determined with Origin software. Differences among different experimental groups were analyzed for statistical significance by means of one-way ANOVA (analysis of variance) with *post hoc* Tukey HSD (honest significant differences) test. A *P* value of <0.05 was considered statistically significant.

### Ethics statement.

This study was carried out in strict accordance with the recommendations of the NACLAR (National Advisory Committee for Laboratory Animal Research) guidelines under the Animal and Birds (Care and Use of Animals for Scientific Purposes) Rules of Singapore. The protocol was approved by the Institutional Animal Care and Use Committee (IACUC) of the Nanyang Technological University of Singapore (number ARF SBS/NIE-A0234). All efforts were made to minimize suffering.
