# Supplementary figures and images for: Differential Spleen Remodeling Associated with Different Levels of Parasite Virulence Controls Disease Outcome in Malaria Parasite Infections
Source: mSphere. 2015 Dec 9;1(1):e00018-15. doi: 10.1128/mSphere.00018-15 (PMC4863626; doi:10.1128/mSphere.00018-15)

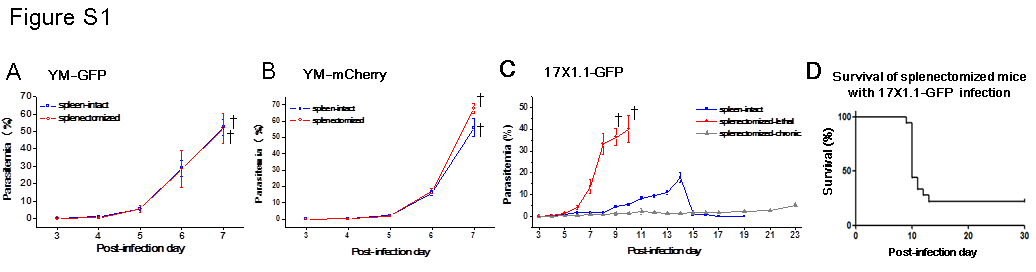

Supplement: Figure S1 [file sph001160037sf1.tif]

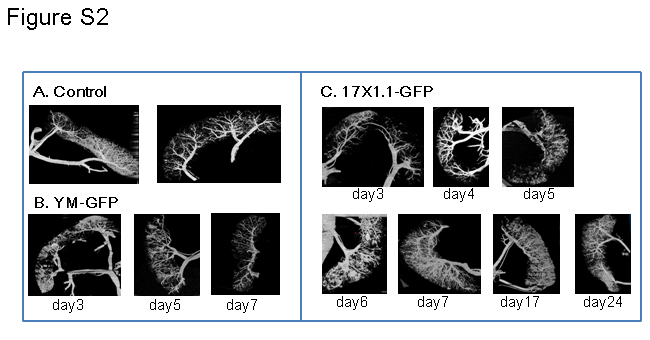

Supplement: Figure S2 [file sph001160037sf2.tif]

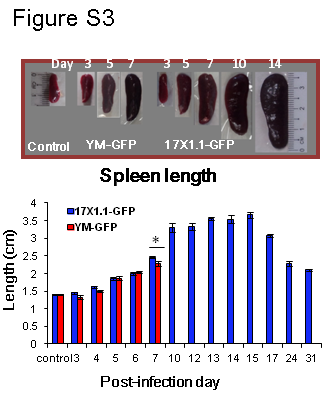

Supplement: Figure S3 [file sph001160037sf3.tif]

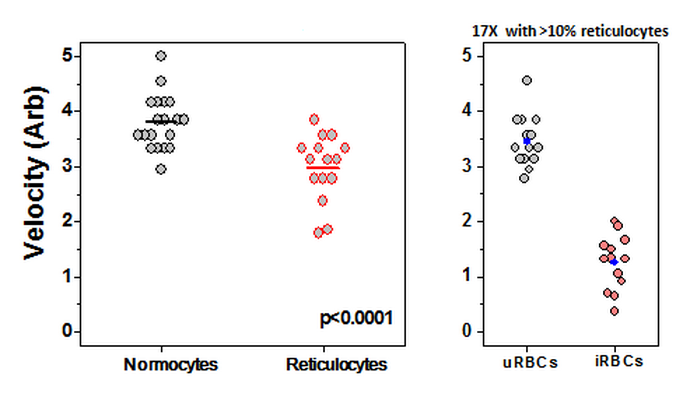

Supplement: Figure S4 [file sph001160037sf4.tif]
